# Global Patch Matching (GPM) for freehand 3D ultrasound reconstruction

**DOI:** 10.1186/s12938-017-0411-2

**Published:** 2017-10-30

**Authors:** Weijian Cong, Jian Yang, Danni Ai, Hong Song, Gang Chen, Xiaohui Liang, Ping Liang, Yongtian Wang

**Affiliations:** 1 Beijing Engineering Research Center of Mixed Reality and Advanced Display, School of Optics and Electronics, Beijing Institute of Technology, Beijing, 100081 China; 2 School of Computer Science and Engineering, Beihang University, Beijing, 100191 China; 3 School of Software, Beijing Institute of Technology, Beijing, 100081 China; 4 Interventional Ultrasound Department, Chinese PLA General Hospital, 28 Fuxing Road, Haidian District, Beijing, 100853 China

**Keywords:** 3D ultrasound reconstruction, Matching patch, Optimal contribution range

## Abstract

**Background:**

3D ultrasound volume reconstruction from B-model ultrasound slices can provide more clearly and intuitive structure of tissue and lesion for the clinician.

**Methods:**

This paper proposes a novel Global Path Matching method for the 3D reconstruction of freehand ultrasound images. The proposed method composes of two main steps: bin-filling scheme and hole-filling strategy. For the bin-filling scheme, this study introduces two operators, including the median absolute deviation and the inter-quartile range absolute deviation, to calculate the invariant features of each voxel in the 3D ultrasound volume. And the best contribution range for each voxel is obtained by calculating the Euclidian distance between current voxel and the voxel with the minimum invariant features. Hence, the intensity of the filling vacant voxel can be obtained by weighted combination of the intensity distribution of pixels in the best contribution range. For the hole-filling strategy, three conditions, including the confidence term, the data term and the gradient term, are designed to calculate the weighting coefficient of the matching patch of the vacant voxel. While the matching patch is obtained by finding patches with the best similarity measure that defined by the three conditions in the whole 3D volume data.

**Results:**

Compared with VNN, PNN, DW, FMM, BI and KR methods, the proposed Global Path Matching method can restore the 3D ultrasound volume with minimum difference.

**Conclusions:**

Experimental results on phantom and clinical data sets demonstrate the effectiveness and robustness of the proposed method for the reconstruction of ultrasound volume.

## Background

Ultrasound, computer tomography (CT) and magnetic resonance (MR) are acknowledged to be the most widely used modern medical imaging techniques in clinical practice [1–3]. Compared with CT and MR, ultrasound imaging has several indispensable advantages, such as fast imaging, no intravenous contrast agents, no ionizing radiation, inexpensive, convenience and so on [4]. Principally, ultrasound detects and magnifies echo acoustic wave that reflects from the human body. By calculating attenuation rates of the acoustic wave, the density distribution of the soft tissue can be effectively deduced [5]. Nowadays, ultrasound is widely used in the abdominal disease diagnosis and treatment for its safe and fast imaging properties. However, 2D ultrasound imaging cannot meet the clinical requirements when the physician needs to obtain the whole 3D anatomic structure of the organs. As the commonly used ultrasound imaging device can only obtain 2D cross-sectional images of the body, it is imperative for the physician to conceive 3D structures of the human body in mind according to their clinical experiments. The approach could affect the accuracy and efficiency for clinical diagnosis and treatment, especially for tissues contain some organ malformations or lesions. Compared with the traditional 2-D ultrasound image, the 3D ultrasound images can provide a more distinct and integrated information of organs and are more suitable for clinical diagnosis and treatment [6, 7].

Generally, there are many ultrasound reconstruction methods, which can be divided into three major categories: 3D probe system, mechanical scanning system and freehand scanning approaches [8]. For the 3D probe system, the 3D ultrasound is directly acquired by a series of dedicated 3D probes with an oscillating mechanism that sweep a predefined region of interested (ROI). This system can generate 3D ultrasound volume through one acquisition. However, it is expensive and incapable of scanning large-volume organs [9]. The mechanical scanning system usually uses the conventional 2D transducer for the image acquisition. During the image acquisition, the transducer is translated and rotated according to a stepping motor, from which the position and orientation information are recorded synchronously in the scanning heads [10–12]. The major problem of the mechanical scanning devices is that its scanning range is constrained by the size and installation axis of the stepping motor [9]. For the freehand scanning approach, conventional 2D probe is integrated with a positioning sensor for labeling the position and orientation of each B-scan image [13]. Hence, as the 2D probe is manipulated by hand in an arbitrarily manner, the freehand system can acquire images at any rotation or orientation of the human body, and it allows the user to manipulate the transducer and view the desired anatomical section freely. A sequence of scanning images are then captured along with its corresponding position and orientation. Freehand 3D ultrasound has received increasing attention for its low-cost, inherent flexibility nature in comparisons with the dedicated 3D probes and mechanical scanning approaches. However, as the orientation and position of the freehand probe are randomly controlled by the user, there is usually a large range of areas are with empty values. Hence, the main task for freehand scanning method is to estimate and interpolate the empty values of 3D volume from the irregularly sampled scanning images, which is still the most challenge task in the 3D reconstruction of ultrasound images.

In general, 3D reconstruction methods of freehand scanning images compose of three major classes [14, 15]: forward method, reverse method and function based method. The forward method can be defined as sequentially scanning and filling the vacant voxel in the 3D ultrasound volume by its neighboring voxels that already have ultrasound values. The key procedures for this method are as follows: first, the predefined 3D volume data is filled by the obtained 2D ultrasound slices according to their acquisition location and orientation. Then, the voxels of the volume data are traversed and iteratively filled by neighboring pixels from the 2D ultrasound slices via some predefined interpolation methods. The simplest method is the voxel nearest neighbor (VNN) [16], which calculates the vacant voxel using its nearest neighboring voxel in the 3D volume data. While the distance weighted (DW) methods [14] interpolate vacant voxel by introducing weighting coefficients that proportional to the distance of the calculating voxels. According to the way of nearest neighboring voxel interpolation, a variety of methods, including squared distance weighted (SDW) [17], adaptive distance weighted (ADW) [18], squared adaptive distance weighted (ASDW) [19], distance weighted median filter (DWMF) [20], adaptive weighted median filter (AWMF) [20] and kernel regression (KR) [21–23] have been utilized for the 3D ultrasound reconstruction. The forward method is intuitive and easy to be implemented. However, for such method, the calculating of each vacant voxel need to traverse the whole volume data once, which is very much time consuming.

On the contrary, the reverse method can be defined as iterative scanning and filling the vacant voxels around the 2D ultrasound slices in the 3D volume data. Generally, there are three major procedures for the reverse methods: first, the 3D ultrasound volume data is filled by the obtained 2D ultrasound slices according to their acquisition location and orientation. Second, each pixel of the ultrasound slice is scanned and the neighboring voxels in the 3D volume are filled according to a specified interpolation method. Third, each voxel of the 3D volume is traversed and the vacant voxels are filled by their neighboring voxels. For such class of method, the simplest way for interpolation is the pixel nearest neighbor (PNN) based methods [24–31], which obtain the intensity of the vacant voxel by interpolating of closing voxels. For the interpolation procedure, the number of voxels utilized for the 3D reconstruction and the weighting coefficient for each utilizing voxels are the two major aspects. It is obvious that the number of vacant voxels is the key to determine the calculation efficiency of the interpolation method. To improve the interpolation efficiency, Toonkum et al. [12] proposed a cyclic Savitzky-Golay filter based method for calculating the priority of each utilizing voxels. This method first inserts the middle frame into the ultrasound slices for minimizing the number of the vacant voxels. From iteratively frame interpolation, the intensity of the vacant region can be estimated. It is important to note that during the interpolation procedure, if the vacant voxels are sequentially filled by their neighboring image pixels, as the pixels are iteratively utilized for the interpolation, the filled region may presents as low differentiability. One promising solution for such a phenomenon is that utilize the neighboring pixels in a certain order that minimizes the blurring effect. Wen et al. [32] proposed a fast marching method (FMM) for the 3D reconstruction, which first detect the boundary of the vacant voxels and then fills the outer voxels in previous and then iteratively fills the inner voxels. Such method can effectively improve the calculation efficiency and interpolation accuracy. The difference between forward approach and reverse approach is that the forward approach searches the vacant voxel in the whole volume data, while the reverse approach searches the vacant voxels around the 2D ultrasound slices in the volume data. Hence, the reverse approach is faster than the forward approach for the 3D reconstruction of ultrasound.

The function based methods usually construct determined relationship between location and its intensity for the vacant voxel in the 3D volume data. Such method usually first interpolates 2D ultrasound slices into the 3D volume data according to their acquisition orientation. Then, the functional relationships between location and intensity distribution of the voxels are constructed according to the filled voxels in the volume data. By statistical analysis, the coefficients of the constructed function are estimated, which hence is utilized to calculate the intensity of the vacant voxels. The most widely used function for the interpolation is the radial basis function (RBF) [33] and Rayleigh [34]. Huang et al. [35] have designed a fast interpolation method for 3D US with sparse scanning based on Bezier interpolation (BI). The advantage of the function based method is that it can obtain high reconstruction precision, but as it needs to estimate the functional coefficients by statistical analysis of all filled voxels, such method is usually with low calculation efficiency.

To improve the reconstruction efficiency and accuracy, this paper proposes a novel Global Path Matching (GPM) method for the 3D reconstruction of freehand ultrasound images. The proposed method composes of two main steps: bin-filling scheme and hole-filling strategy. For the bin-filling scheme, the vacant voxels around each pixel of the 2D ultrasound image in the 3D volume is filled by weighted combination of its neighboring pixels that with eigenvalue less than a predefined threshold. It is commonly known that there are large amounts of random noise in the ultrasound image, and the existence of the noise may greatly interfere the 3D reconstruction accuracy. To improve the interpolation accuracy, this study introduces two operators, including the median absolute deviation (MAD) and the inter-quartile range absolute deviation (IQRAD) [39], to calculate the invariant features of each voxel in the 3D ultrasound volume. As the two features are insensitive to the ultrasound noise, the feature response of each voxel can be defined as the weighted combination of the MAD and IQRAD. Hence, the best contribution range for current calculating voxel can be defined as the Euclidian distance between the pixel with minimum feature response and current voxel. Once the best contribution range is obtained, the intensity of current vacant voxel can be obtained by weighted combination of the pixels within the best contribution range, for which the weights are inverse proportional to the distance between the vacant voxel and the pixels in the best contribution range. The merit of the proposed bin-filing scheme is as follows: (a) the boundary vacant voxel is determined by the neighboring pixels within the best contribution distance, which hence can effectively minimize the blurring effects. (b) The best contribution distance is automatically determined by the invariant features of the pixels, which is more robust and objective than the threshold based methods. (c) The intensity of the vacant voxel is determined by the weighted combination of pixels within the best contribution distance, for which the weights are inverse proportional to the Euclidian distance between current voxel the vacant voxel. Hence, the intensity of the vacant voxel is determined by all its neighboring voxels, which can effectively smooth the whole volume data.

The hole filling strategy is defined to fill the vacant voxels in the 3D ultrasound volume data through finding the best matching patch in the whole 3D ultrasound volume data. The vacant voxels in the 3D ultrasound volume are first marked through traverse the whole volume data, and the vacant region are labeled by calculating connecting relationship between neighboring vacant voxels. Then, three conditions, including the confidence term, the data term and the gradient term, are designed to calculate the weighting coefficient of the matching patch. More in detail, the confidence term represents the gray scale distribution, and the data term is utilized to represent contour information, while the gradient term is utilized to represent the intensity variation. This study assumes that the differences between the matched patches should be the minimum for all the three condition terms. Based on this assumption, the best matching patch for the vacant voxel is obtained by finding patches with identical combined matching conditions in the whole 3D volume data. Then, the intensity of the vacant voxel is replaced by voxels in the matching patch, for which the voxels in the patch are weighted contributed to the boundary voxels. Finally, the whole volume data is filled by iteratively repeating the above processing procedures. The merits of the designed 3D hole filling strategy method are as follows: (a) the filling patch of the vacant region is obtained by searching the best matching in the whole volume data, which hence can effectively improve the reconstruction accuracy. (b) As the hole filling is initiated from the boundary region with maximum weighting coefficients, hence, the filling process can achieve high reconstruction accuracy and effectively preserve the continuity of the texture information of the whole ultrasound volume data.

## Methods

**Fig. 1:**
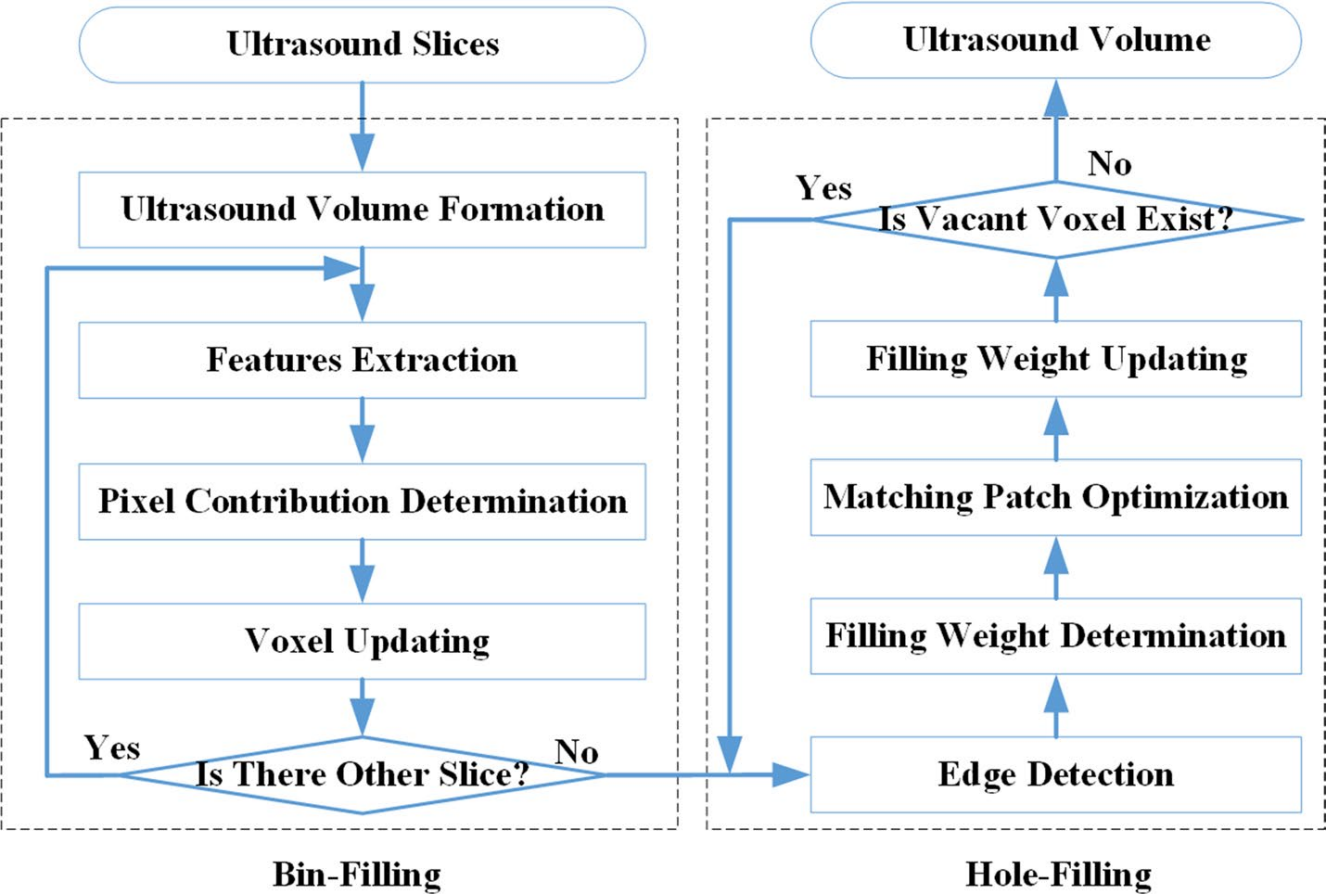
Outline of the ultrasound reconstruction algorithm

In this paper, the ultrasound reconstruction is performed in two major stages, as can be seen in Fig. 1. In the bin-filling scheme, we first insert every ultrasound slices into the ultrasound volume according to the location and orientation of the ultrasound slice. And the best contribution range and contribution distance are calculated by the predefined invariant feature of each pixel. Then, the intensity of each voxel in ultrasound volume can be determined and updated. In the 3D hole-filling strategy, we first detect the edge of vacant region and calculate the filling weight of all voxels on the edges. And the best matching patch for the vacant voxel is obtained by finding patches in the whole volume, and the intensity of the vacant voxel is replaced by voxels in the matching patch. Then, the filling weight of the voxel on the edge is updated, and all the vacant voxels are iteratively filled by repeating the above processing procedures.

### Image acquisition system

The freehand 3D ultrasound imaging acquisition system consists of three main devices: a conventional 2D ultrasound scanner (DC-7, Mindray Medical International Ltd., Shenzhen, China), an electromagnetic spatial sensing device (Aurora, NDI Ontario, Canada), and a workstation with custom-designed software for data acquisition, volume reconstruction, and visualization. The receiver of the spatial sensing device is attached to the 4.5 MHz hand-held probe of the ultrasound scanner. The spatial information (location and orientation) between the receiver and transmitter is recorded and transferred from the Aurora system control unit to the workstation through a USB port.

During data acquisition, spatial data and digitalized 2D ultrasound images are simultaneously recorded and collected. Since the devices for the collection of 2D ultrasound and spatial data are independent, the temporal delay between the two data streams cannot be avoided. Meanwhile, the spatial relationship between the ultrasound image plane and magnetic position sensor needs to be determined. The temporal and spatial relationships for the designed freehand 3D ultrasound imaging system are calibrated by the methods presented in [36, 37].

**Fig. 2:**
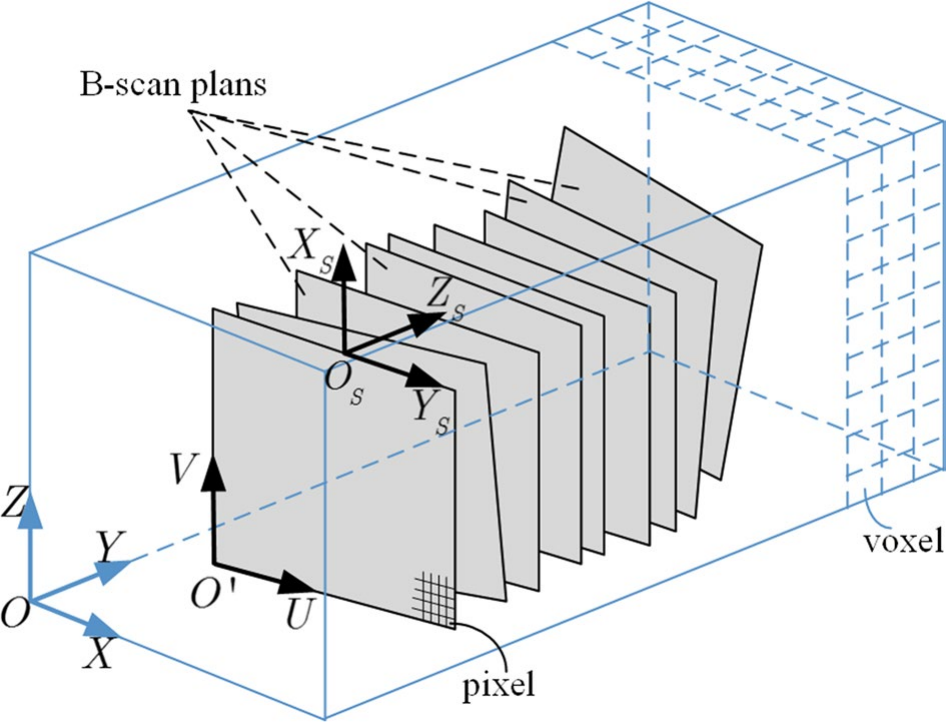
Relationship between ultrasound slices and 3D volume

Generally, there are three coordinates in the reconstruction system: the coordinate of the 3D ultrasound volume ($O - XYZ$), the coordinate of the 2D ultrasound slices ($O^{\prime} - UV$) and the coordinate of the spatial sensing device ($O_{S} - X_{S} Y_{S} Z_{S}$). The transformation between $O^{\prime} - UV$ and $O_{S} - X_{S} Y_{S} Z_{S}$ can be determined by the information that provided by the spatial sensing device. Suppose $(u_{ji} ,v_{ji} )$ is the ith pixel in the jth ultrasound slice, $(N_{jU} ,N_{jV} )$ is the size of the jth ultrasound slice, $(S_{jU} ,S_{jV} )$ is the image spacing of the jth ultrasound slice, $(X_{{S_{j} }} ,Y_{{S_{j} }} ,Z_{{S_{j} }} )$ is the spatial position of the jth ultrasound slice with respect to the information obtained from the spatial sensing device. As illustrated in Fig. 2, the position $(X_{{S_{ji} }} ,Y_{{S_{ji} }} ,Z_{{S_{ji} }} )$ of the pixel in the coordinate $O_{S} - X_{S} Y_{S} Z_{S}$ system can be obtained by the following equations:

1 $$\left\{ \begin{aligned} & X_{{S_{ji} }} = X_{{S_{j} }} + \left(u_{ji} - \frac{{N_{jU} }}{2}\right) \times S_{jU} \\ & Y_{{S_{ji} }} = Y_{{S_{j} }} - (N_{jV} - v_{ji} ) \times S_{jV} \\ & Z_{{S_{ji} }} = Z_{{S_{j} }} \\ \end{aligned} \right.$$

Let R, t and r represent the parameterized transformation between coordinates $O - XYZ$ and $O_{S} - X_{S} Y_{S} Z_{S}$, then, the coordinate of $(X_{j} ,Y_{j} ,Z_{j} )$ in the system $O - XYZ$ corresponding to the point $(X_{{S_{j} }} ,Y_{{S_{j} }} ,Z_{{S_{j} }} )$ in the coordinate system $O_{S} - X_{S} Y_{S} Z_{S}$ can be obtained as follows:

2 $$\left[ \begin{aligned} X_{j} \hfill \\ Y_{j} \hfill \\ Z_{j} \hfill \\ \end{aligned} \right] = \left[ {\begin{array}{*{20}c} R & T \\ 0 & r \\ \end{array} } \right]\left[ \begin{aligned} X_{{S_{j} }} \hfill \\ Y_{{S_{j} }} \hfill \\ Z_{{S_{j} }} \hfill \\ \end{aligned} \right]$$

As the location and orientation of each ultrasound image are recorded by the electromagnetic spatial sensing device, the combined 3D ultrasound volume hence can be obtained by transform each pixel of the 2D ultrasound image into the normalized coordinate.

### Bin-filling scheme

#### Features extraction

The bin-filling scheme is designed to fill the 3D volume data by the ultrasound slices according to its acquisition location and orientation. As the obtained image slices are discrete and may intersect with each other, the aim of the bin-filling is to fill the vacant voxels by their neighboring pixels in the 2D ultrasound images. As there are large amounts of noise in the ultrasound image, which may interfere the 3D reconstruction accuracy. Hence, this study introduces the median absolute deviation (MAD) [38] and the inter-quartile range absolute deviation [39] (IQRAD) to calculate the invariant property of pixels in the ultrasound image, which aims to find the unique representation of the local patch that has minimum interference of the noise.

The MAD is utilized to represent the intensity difference between current and its neighboring pixels in a predefined patch. Suppose there are N pixels surrounding the calculating pixel $u_{i}$ in the predefined patch, $u_{i}^{j} ,(j \in N)$ is the jth neighboring pixel of $u_{i}$, while $I(u_{i} )$ represents the intensity of $u_{i}$. Let $u_{i}^{mid}$ represents the pixel with median intensity value in the neighboring patch of $u_{i}$, then, the MAD of $u_{i}$ can be calculated as follows:

3 $$MAD(u_{i} ): = abs(I(u_{i}^{mid} ) - I(u_{i} ))$$

IQRAD represents the average intensity difference of pixel $u_{i}$ in its neighboring patch. Suppose $I_{\text{max} } (u_{i} )$ and $I_{\text{min} } (u_{i} )$ represent maximum and minimum intensities in the neighboring patch of $u_{i}$, then, pixels with intensity in the ranges of 25 to 75% of the maximum intensity can be calculated as follows:

4 $$\begin{aligned} u_{i}^{25\% \to 75\% } \subset \left[I_{\text{min} } (u_{i} ) + 0.25 \times (I_{\text{max} } (u_{i} ) - I_{\text{min}} (u_{i} )),\right. \\ \left. I_{\text{min} } (u_{i} ) + 0.75 \times (I_{\text{max} } (u_{i} ) - I_{\text{min} } (u_{i} ))\right] \end{aligned}$$

Hence, the IQRAD of the pixel $u_{i}$ can be defined as follows:

5 $$IQRAD(u_{i} ): = \sum\limits_{j}^{{}} {(I(u_{i}^{j} ) - I(u_{i} ))} /n,u_{i}^{j} \in u_{i}^{25\% \to 75\% }$$

where n is the number of pixels in the ranges of 25 to 75% of the maximum intensity.

It can be seen that MAD and IQRAD both utilize all pixels in the patch to calculate the property of $u_{i}$, which hence are very robust to noise and outliers. To calculate the unique property of each patch, this study combines MAD and IQRAD as follows:

6 $$H(u_{i} ) = \alpha \times MAD(u_{i} ) + \beta \times IQRAD(u_{i} ),$$

where $\alpha$ and $\beta$ are weighting coefficients of MAD and IQRAD, and they can be determined by the following equations:

7 $$\alpha\; { = }\;\frac{{\frac{3}{4}(N{ + }1)}}{{N + n{ + }1}},\;\beta\; { = }\;\frac{{\frac{1}{4}(N + 1){ + }n}}{N + n + 1}.$$

#### Pixel contribution determination

It is obvious that different patch size may lead to different value of the combined response of $H(u_{i} )$, while the robust patch should have the minimum $H(u_{i} )$. Hence, this study adaptively calculates $H(u_{i} )$ in the predefined ranges that determined by the distance between $u_{i}$ and its neighboring pixels. In this study, the distances between current pixel and its neighboring pixels are indexed as eight classes. Then, the best contribution range $\varOmega (u_{i} )$ of the pixel $u_{i}$ in the patch can be defined as:

8 $$\varOmega (u_{i} ) = {\text{min(}}H_{k} (u_{i} ) ) , \quad { }k = 1 , 2 ,\ldots , 8$$

It is obvious that the best contribution range depicts that the pixels in this range have the minimum intensity differences with the central pixel. Hence, the best contribution distance can be calculated as follows:

9 $$d(u_{i} ) = max_{{v \in \varOmega (u_{i} )}} (d(u_{i} ,v)),$$

where $d(u_{i} ,v)$ represents the Euclidian distance between $u_{i}$ and $v$ in the best contribution range. Once the best contribution range is determined, the local intensity response of $H(u_{i} )$ can be obtained by calculating the integrated response of each pixel to $u_{i}$ in the spherical ball with radius of $d(u_{i} )$. While the relative response of two pixels in the spherical ball can be calculated by the following equation:

10 $${\text{I(}}u_{i} ,x_{j} ) = I(u_{i} )/d(u_{i} ,x_{j} ),$$

where $I(u_{i} )$ is the intensity of the pixel $u_{i}$.

#### Voxel updating

Hence, the vacant voxel in the 3D ultrasound volume can be obtained by integrating the relative responses of each pixel in the best contribution range, and we have the following equation:

11 $$V(x_{j} )\;{ = }\;\frac{{\sum\limits_{i = 1}^{n} {I(u_{i} ,x_{j} )} }}{{\sum\limits_{i = 1}^{n} {D(u_{i} ,x_{j} )} }} = \frac{{\sum\limits_{i = 1}^{n} {I(u_{i} )/D(u_{i} ,x_{j} )} }}{{\sum\limits_{i = 1}^{n} {D(u_{i} ,x_{j} )} }}.$$

Compared with the other interpolation method, the propose bin-filling method weighted combines the pixels in the best contribution range centered at current pixel, which guarantees that the interpolated voxel is most similar and has minimum variations to its neighboring pixels.

### Hole-filling strategy

After the bin-filling process, the 3D volume data is filled with the 2D ultrasound slices according to their imaging orientation and location. As the obtained ultrasound slices are not continuous, it is obvious that there are a lot of holes and empty voxels in the 3D volume data. The goal of the hole-filling stage is to fill the gaps using available information from its surrounding known voxels. Most of current methods usually utilized the neighboring voxels in the 3D ultrasound volume instead of the global information of the volume data [40–44]. To obtain the best filling effect of the 3D volume data, this study proposed a novel global patch matching method, which search the most similar voxels in the whole ultrasound volume data.

The basic idea of the proposed hole filling method is that for any vacant voxel in the 3D volume data, the filling intensity of the voxel is obtained by searching a patch that is with the most similar intensity distribution around the vacant voxel in the whole volume data. First, for the vacant voxel, we construct a rectangular patch by setting the vacant voxel as the center. Then, priority weight of the filling is calculated for each voxel in the patch according to the vacant voxel number, isophotes of the center voxel and the gradient distribution of the patch. Second, the hole-filling is initiated from the voxel with maximum priority weights. The best matching patch to the patch centered at current voxel is iteratively scanned in the whole volume data by maximizing the similarity measure. Third, the vacant voxels of the current patch are customized by the corresponding voxels in the best matching patch. Then, the priority weight of the whole image is recalculated. The filling process repeats the above procedures until all the vacant voxels are assigned with intensity values.

**Fig. 3:**
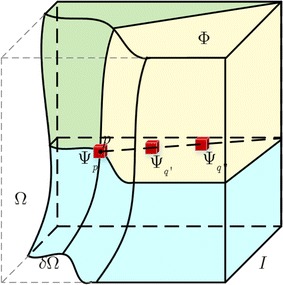
The hole filling method based on image texture

Let I denotes the given input image, $\varOmega$ denotes the target region that needs to be filled (the dashed area), and $\delta \varOmega$ denotes the surface of the region $\varOmega$. Then, the source region $\varPhi$ can be calculated as the entire image minus the target region $(\varPhi = {\rm I} - \varOmega )$, as can be seen in Fig. 3. In this figure, the regions of blue, green and yellow represent different intensity values in the ultrasound volume. p represents the border pixel for the three regions, $\varPsi_{p}$ represents the patch centered at p, while $\varPsi_{q'}$ and $\varPsi_{q''}$ represent the best matching two patches with $\varPsi_{p}$. Then, the filling value of $\varPsi_{p}$ should be determined by $\varPsi_{{q^{\prime}}}$ and $\varPsi_{{q^{\prime\prime}}}$ that rest on the border of the neighboring patches. The detail filling processes are as follows:

#### Filling weight determination

For the ultrasound reconstruction method, it is obvious that different repairing order may lead to different repairing accuracy. Hence, the repairing order is the priority step to be determined before the proceed of the repairing process. As the proposed method searches the most similar patches in all the voxels filled with intensity values, there are three main aspects need to be taken into consideration for the design of repairing process: first, if there are more voxels filled with intensity values, the global searching procedure may obtain more accurate repairing accuracy. Hence, if a patch with more filled voxels, the filling priority of the patch will be higher than the other voxels. Second, the isophotes is usually taken to represent the continuousness and smoothness. Hence, to guarantee the continuity and smoothness of the ultrasound volume, the voxel with higher filling priority should rest on the isolux curve. Third, if the gradient of a voxel is larger than the others, the edge points can usually be identified. Hence, the patch with the larger summary of the gradient should be with higher priority. Based on the above analysis, three terms, including confident term $C(p)$, data term $D(p)$ and gradient term $G(p)$, are included to calculate the priority weight of the center point $p$ of the patch $\varPsi_{p}$.

The confident term $C(p)$ is utilized to measure the intensity distributions of existing voxels. It is commonly known that if there are more voxel existed in the whole ultrasound volume, the patch matching will be more accurate. Also, if there are more voxels in the matching patch, the global patch searching will be more accurate. Hence, the confident term can be defined by all the intensity accumulation divided by the size of the patch. We have:

12 $$C(p) = \frac{{\sum\nolimits_{{q \in \varPsi_{p} \cap (I - \varOmega )}} {C(q)} }}{{|\varPsi_{p} |}}.$$

The data term $D(p)$ is utilized to measure the smoothness of the region $\varOmega$ that need to be filled. It is obvious that the value of $D(p)$ is proportional to the isophotes of the intensity, which depicts the propagation information of the ultrasound structures. The involving of the data term guarantees that the path with linear structure will be repaired in prior, hence, the filling structure can be attained linear connection. Broken lines tend to connect, thus it realizes the connectivity principle of the vision psychology [45–47]. Let $\alpha$ represents the normalization factor, $n_{p}$ denotes a unit vector orthogonal to the front $\delta \varOmega$ in the point $p$, and $\bot$ denotes the orthogonal operator. The data term $D(p)$ can be calculated by the following equation:

13 $$D(p) = \frac{{|\nabla I_{p}^{ \bot } \cdot n_{p} |}}{\alpha }$$

The gradient term $G(p)$ is utilized to represent the variability of the intensity in the patch $\varPsi_{p}$. For the vacant region of the ultrasound volume, if the edge section is deficient, the edge section needs to be repaired preferentially. And edge information can be effectively detected by the gradient of the patch. Let $I_{qx}$, $I_{qy}$ and $I_{qz}$ represent the three components of the gradient vector of point $p$ in the directions of x, y and z, respectively. Then, the gradient term can be calculated by the following equation:

14 $$G(p) = \frac{{\sum\nolimits_{{q \in \varPsi_{p} \cap ({\rm I} - \varOmega )}} {\sqrt {I_{qx}^{2} + I_{qy}^{2} + I_{qz}^{2} } } }}{{|\varPsi_{p} \cap ({\rm I} - \varOmega )|}}.$$

So far, the repairing priority weight of the voxel $p$ can be calculated by combining the confident term, data term and gradient term. We have:

15 $$P(p) = C(p)D(p)G(p).$$

Once the priority weights of all the vacant voxels are calculated, the hole filling process can be started at the patch $\varPsi_{{\hat{p}}}$ with highest priority.

#### Matching patch optimization

For the vacant voxel with largest priority weight, the intensity utilized to fill the vacant voxel is obtained by finding patches with the largest intensity similarity in the whole ultrasound volume. And the similarity is defined by the inverse of the absolute intensity difference of the two comparing patches. Hence, we have the following equation:

16 $$\varPsi_{{\hat{q}}} = \arg \mathop {\hbox{min} }\limits_{{\varPsi_{q} \in \varPhi }} d(\varPsi_{{\overset{\lower0.5em\hbox{$\smash{\scriptscriptstyle\frown}$}}{p} }} ,\varPsi_{q} ),$$

where $$\varPsi_{q}$$ is the patch that needed to be filled, $$\varPsi_{{\overset{\lower0.5em\hbox{$\smash{\scriptscriptstyle\frown}$}}{p} }}$$ is any patch with identical size of $$\varPsi_{q}$$ in the ultrasound volume. $$d(\varPsi_{a} ,\varPsi_{b} )$$ represents the absolute intensity difference of the two patches. If the source exemplar $$\varPsi_{{\hat{q}}}$$ is determined, the value of each pixel that to be filled $$p^{\prime}|p^{\prime} \in \varPsi_{{\hat{p} \cap \varOmega }}$$ is copied from its corresponding position inside $$\varPsi_{{\hat{q}}}$$. Iteratively repeats the filling process, the structure and texture information can be gradually propagated from the source $$\varPhi$$ to the target region $$\varOmega$$.

#### Filling weight updating

Once a vacant patch is filled with new pixel value, the distribution of the vacant region is changed. Hence, the priority weight of each vacant voxel in the whole ultrasound volume needs to be recalculated so as to find the best region that needs to be filled. For the proposed method, we only need to recalculate the priority weights of the neighboring voxels of the previous filled patch. Then, the filling process iteratively repeats until all the vacant voxels are filled. The pseudocode of the proposed algorithm can be found in Table 1.

**Table 1:**
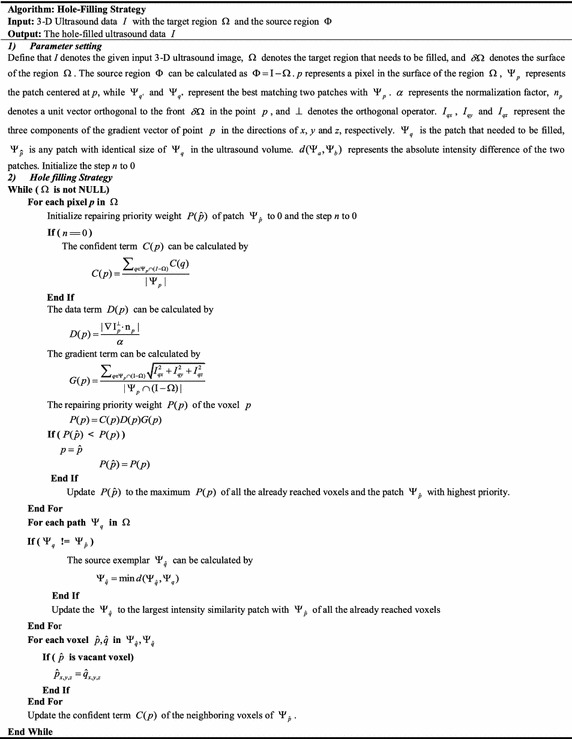
Pseudocode for the proposed GPM algorithm

Compared with traditional ultrasound reconstruction method, the proposed method finds the best matching patch from the whole ultrasound volume, which can effectively prevent the over smoothing of the image. Moreover, the proposed method repairs the patch with more filled voxels and the patch with apparent gradient variations, which hence can obtain accurate reconstruction accuracy.

## Experimental results

To evaluate the performance of the proposed GPM method for the 3D reconstruction of freehand ultrasound, a series of experiments is designed and tested on phantom data and in vivo scanned ultrasound in clinical practice. The proposed method is compared with the up-to-date algorithms, including VNN [16], PNN [14], DW [14], FMM [32], KR [21–23] and BI [35] methods, and the accuracies of the comparing methods are evaluated via average interpolation error of the vacant voxels. The entire algorithm was implemented by the C++ programming language under the Linux platform, and all the experiments were performed on a relative low-cost desktop computer with an i7-4770 processor and 16G memory.

In this study, three groups of data sets, including ultrasound volume generated from phantom data (data 1), in vivo data set (data 2), are employed for the experiments. The ultrasound volume of a simulation model is generated from a realistic brain phantom created from polyvinyl alcohol cryogel (PVA-C) by Sean Jy-Shyang Chen et al. [48]. PVA-C has been widely used for validating image processing methods, including segmentation, reconstruction, registration, and denoising, for its mechanically similar to the soft tissues of the human body. The phantom was cast into a mold designed from the left hemisphere of the Colin27 brain data set and contains deep sulci, a complete insular region, and an anatomically accurate left ventricle. The author released the CT, MRI and ultrasound images of the phantom. All the volume data sets are with the resolution of 339 × 299 × 115, and corresponding imaging angles of ultrasound. The in vivo data set (data 1) is obtained from the Medical Imaging Group, University of Cambridge [49]. The data set include 135 frames B-model ultrasound slices and spatial information.

In additional to the visual assessment, we measure the performance of the various reconstruction algorithms through computing the average intensity difference as defined as follows [14]:

17 $$V = \frac{1}{N}\sum\limits_{n = 1}^{N} {|\nu_{n} - \nu^{\prime}_{n} |} ,$$

where $N$ is the total number of voxels within the reconstructed volume. While $\nu$ and $\nu^{\prime}$ are the gray value of voxel $n$ of the reconstructed and the phantom volume data sets, respectively. It is obvious that a smaller $V$ indicates a better performance of the reconstruction algorithm.

### Evaluation of hole-filling results

**Fig. 4:**
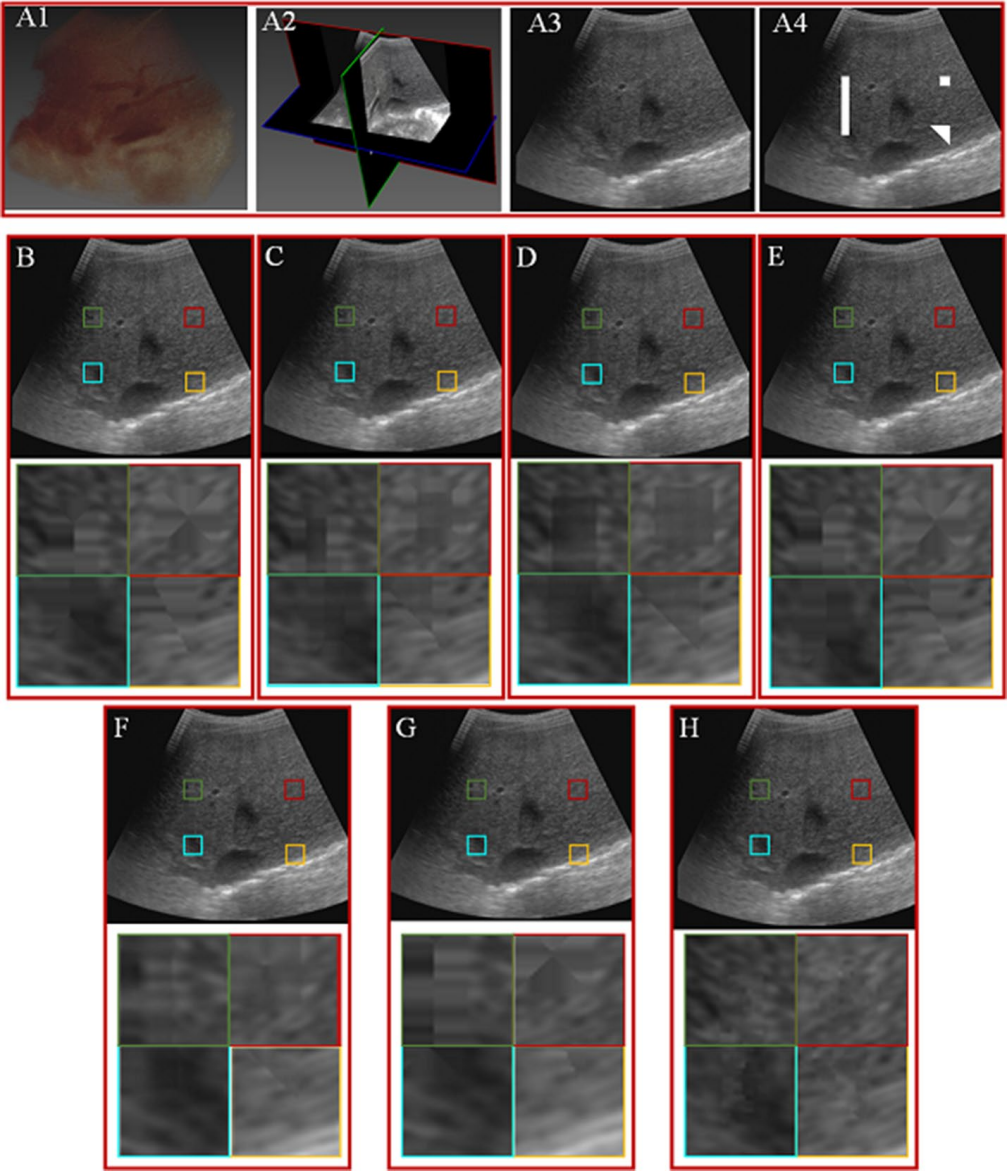
Comparison of different reconstruction methods over data 1. **A1** 3D rendering of ultrasound volume. **A2** Cross-section of the ultrasound volume. **A3** Ultrasound slice. **A4** Ultrasound slice with holes. **B**–**H** Correspond to reconstruction results of the VNN, PNN, DW, FMM, KR, BI and the proposed GPM methods. The third column shows the magnified regions of interest corresponding to the second column

This part is designed to test the effectiveness of the proposed hole-filling algorithm by comparing with the up-to-date algorithms, including VNN, PNN, DW, FMM, KR and BI. The experiments utilize data set 2, and three predefined sizes of volumes, including two cubes (20 × 20 × 20 and 15 × 50 × 20) and a tetrahedron (as can be seen in Fig. 4) are removed from the original data set. Figure 4 demonstrates the reconstruction results of the VNN, PNN, DW, FMM, KR, BI and the proposed GPM methods. Figure 4A1 gives the volume rendering result of the utilized 3D ultrasound data set, from which the main arteries can be effectively visualized. Figure 4A2 shows the cross-section of the ultrasound volume, while Fig. 4A3 shows one single slice of the ultrasound volume. Figure 4A4 shows the ultrasound slice with predesigned holes. Figure 4B–H give the reconstruction results of the VNN, PNN, DW, FMM, KR, BI and GPM methods. It can be seen that all the five methods capable of filling the holes by intensities similar to their neighboring pixels. However, from the enlarged views of the filled hole, it can be seen that there are apparent boundaries for the results of VNN, PNN and KR methods. Compared with VNN and PNN methods, there are no obvious boundaries for the filled and the neighboring regions for the DW method. The filled region of the FMM and BI method seems clearer than that of the DW method. Compared with the other methods, it can be seen that the proposed GPM method not only effectively preserves the detail of the filling region, but also, the filled regions are naturally mixed with the neighboring pixels. There are no distinct boundaries for the filling regions. Obvious, the proposed GPM method can obtain the best reconstruction results for all the comparing methods.

Table 2 compares the reconstruction errors of the NN, PNN, DW, FMM and the proposed GPM methods with respect to mean error and standard deviation. From the table, it can be seen that the mean errors for NN, PNN, DW, FMM, KR, BI and GPM are 7.319, 7.144, 6.926, 6.381, 5.915, 6.094 and 5.368, respectively. While the standard deviation for NN, PNN, DW, FMM, KR, BI and GPM are 8.163, 7.842, 7.167, 7.031, 6.511, 6.606 and 5.655, respectively. Obvious, NN and PNN produce worse results than the other three methods. We can conclude that the nearest neighbor interpolated method utilized by VNN and PNN induces the reconstruction error. For all the three classes of patches, it can be seen that the proposed GPM method steadily produces the minimum reconstruction error. For the triangle, rectangle and square patches, the reconstruction errors for the GPM method are 4.517, 6.061 and 5.803, respectively. While for all the comparing methods, the proposed GPM method produces the minimum reconstruction error, for which the mean and standard deviation are 5.368 and 5.655, respectively.

**Table 2:** Comparing of reconstruction errors for all the five methods over three different vacant patches

Method	Triangle patch				Rectangle patch				Square patch				Total			
	Min	Max	Mean	SD	Min	Max	Mean	SD	Min	Max	Mean	SD	Min	Max	Mean	SD
VNN	1	48	8.376	9.649	1	46	6.382	6.762	1	41	7.122	7.484	1.000	45.000	7.319	8.163
PNN	1	42	7.556	9.365	2	43	7.569	7.673	1	44	6.799	6.993	1.333	43.000	7.144	7.842
DW	0	51	7.192	7.373	1	39	6.818	6.940	1	41	7.313	7.651	0.667	43.667	6.926	7.167
FMM	1	39	5.927	7.660	0	53	6.497	6.515	2	45	6.438	6.653	1.000	45.667	6.381	7.031
KR	1	43	5.219	6.643	1	46	6.416	6.461	1	51	6.109	6.428	1.000	46.667	5.915	6.511
BI	0	49	5.683	6.835	1	44	6.275	6.406	0	46	6.323	6.576	0.667	46.333	6.094	6.606
GPM	1	40	4.517	5.367	1	43	6.061	6.132	1	46	5.803	5.868	1.000	43.000	5.368	5.655

### Quantification of slice repairing results

This part is designed to evaluate the performance of vacant slice repairing for all the comparing methods. The experiments utilize data 2, for which we randomly delete one to eight continuous slices from the ultrasound volume data. Totally, six patches are removed from the data set. Then, VNN, PNN, DW, FMM, KR, BI and GPM methods are utilized to reconstruct the vacant voxels. As the original data contains the vacant voxels that to be reconstructed, the performance of the comparing methods hence can be effectively evaluated.

**Fig. 5:**
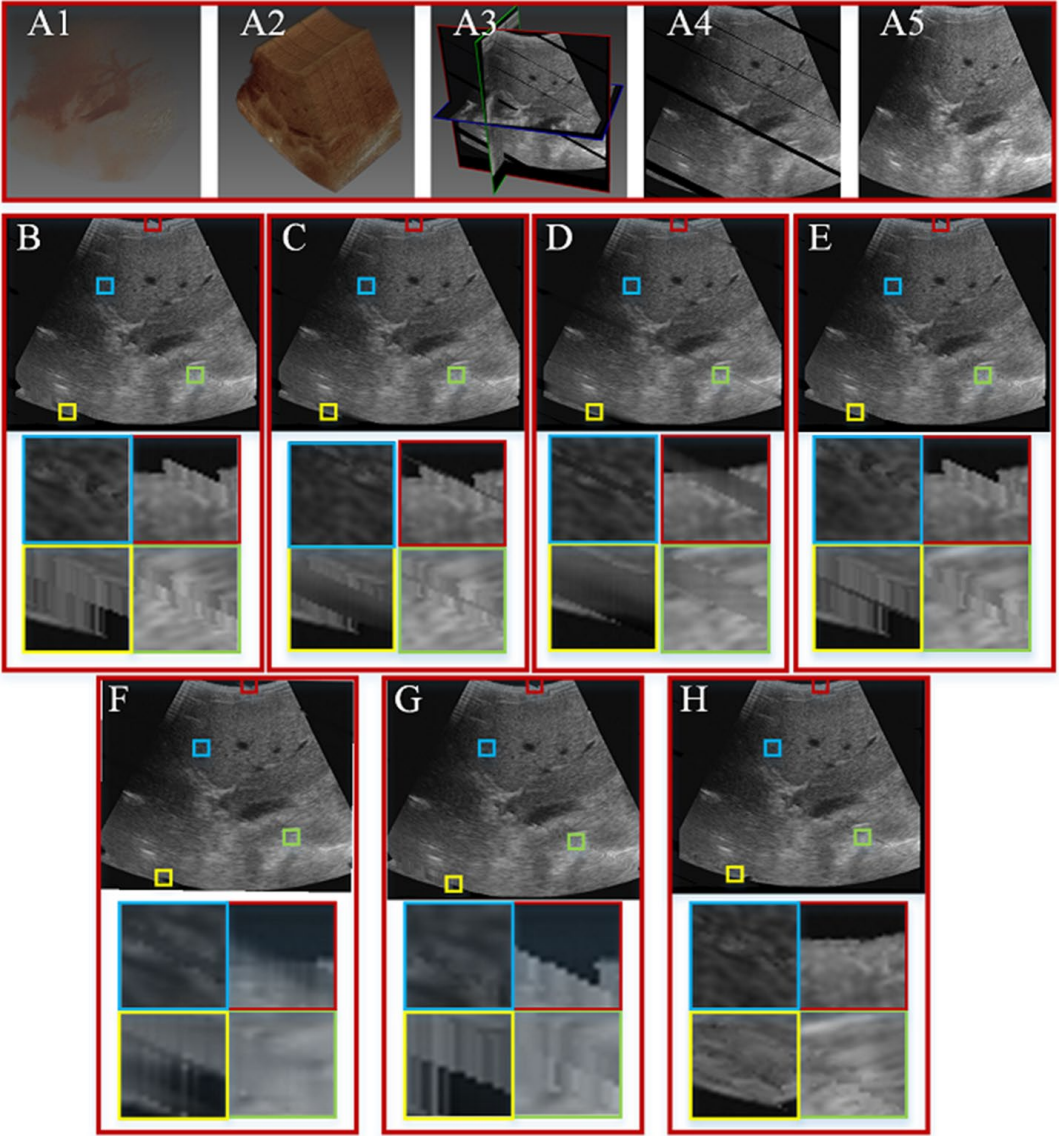
Comparison of different reconstruction results for data 2. **A1** Volume rendering of the ultrasound data. **A2** Ultrasound volume with vacant patches. **A3** Cross-sections of the ultrasound volume data. **A4** Ultrasound slice with vacant patches. **A5** Original ultrasound slice. **B**–**H** Reconstruction results of the VNN, PNN, DW, FMM, KR, BI and GPM methods. The third column shows the magnified regions of interest corresponding to the second column

Figure 5 shows the reconstruction results of the VNN, PNN, DW, FMM, KR, BI and GPM methods on data 2. Figure 5A1 shows volume rendering of the ultrasound data, while Fig. 5A2 shows the ultrasound volume with vacant patches. Figure 5A3 is the cross-sections of the ultrasound volume, Fig. 5A4 provides the ultrasound slice with vacant patches, and Fig. 5A5 shows the original ultrasound slice. Figure 5B–H show the reconstruction results of the VNN, PNN, DW, FMM, KR, BI and GPM methods. The third column shows the magnified regions of interest corresponding to the second column. From the figure, it can be seen that all methods capable of repairing the vacant patches by similar intensities that approach to their neighboring pixels. However, the boundaries of the vacant area can be clearly identified for the VNN, PNN, and KR methods, and the filled regions seem to have the same gray scale distribution. Comparably, the boundaries of the reconstructed region are not as clear as that of the VNN, PNN and KR methods. However, the reconstructed regions of the DW method seems more blurred and homogeneous than that of the VNN, PNN, KR methods. While for the FMM and BI method, there are numerous stripe-like patches in the reconstructed are. Also, the boundary for the reconstructed region can be clearly visualized for the patches with a large number of vacant voxels. Compared with the VNN, PNN and DW methods, the blurred areas reduce clearly, there are still protruding and recessed areas can be visualized. In all the comparing methods, it is obvious that the proposed GPM method produces the best reconstruction results. For the GPM method, the reconstructed region is seamlessly merged with the neighboring pixels. And even the boundary is hardly identified in the reconstructed regions.

From the above experiments, it can be seen that the larger the vacant region is, the larger the reconstruction error will be. To quantify the performance of the reconstruction methods, we randomly delete one to eight continuous slices from the ultrasound volume data. Then, all the five evaluating methods, including VNN, PNN, DW, FMM, KR, BI and GPM, are utilized to reconstruct the ultrasound data. Table 3 provides the reconstruction errors for all the evaluating methods. It can be seen that the reconstruction error increase with the increase of the removing slices for all the five reconstruction methods. From one to eight removing slices, the reconstruction errors increase about 66.8, 68.5, 74.0, 71.5, 61.8, 65.3 and 55.4% for the VNN, PNN, DW, FMM, KR, BI and GPM methods, respectively. From the table, it also can be seen that error decreases regularly from the VNN to the GPM method. It is obvious that all the comparing methods are very stable for the reconstruction of the vacant region in the ultrasound volume data. Comparably, the proposed GPM method is the best method, for which the minimum reconstruction error is generated for each level of vacant patches.

**Table 3:** Comparison of the reconstruction results for different methods over different number of vacant slices

Number of vacant slices	VNN	PNN	DW	FMM	KR	BI	GPM
1							
Mean	8.011	7.793	6.818	5.803	5.694	5.719	5.617
SD	8.144	7.750	6.861	5.896	5.835	5.840	5.820
2							
Mean	8.425	8.493	7.972	6.509	6.437	6.513	6.360
SD	8.461	8.439	8.055	6.835	6.541	6.658	6.396
3							
Mean	9.331	9.152	8.700	7.519	7.262	7.380	7.058
SD	9.634	9.442	8.707	7.612	7.355	7.557	7.285
4							
Mean	10.689	10.283	9.500	8.277	7.729	7.905	7.539
SD	10.616	10.257	9.463	8.227	7.816	8.044	7.773
5							
Mean	11.844	11.109	10.319	9.063	8.479	8.616	8.145
SD	11.867	11.172	10.269	8.983	8.650	8.843	8.155
6							
Mean	12.604	12.211	11.343	9.618	8.881	9.272	8.554
SD	12.897	12.427	11.326	9.742	9.026	9.533	8.511
7							
Mean	13.151	12.817	11.642	9.719	8.945	9.252	8.629
SD	13.117	12.727	11.569	9.979	9.177	9.510	8.729
8							
Mean	13.365	13.128	11.863	9.953	9.345	9.618	8.727
SD	13.385	13.308	12.153	9.973	9.508	9.762	9.033

**Fig. 6:**
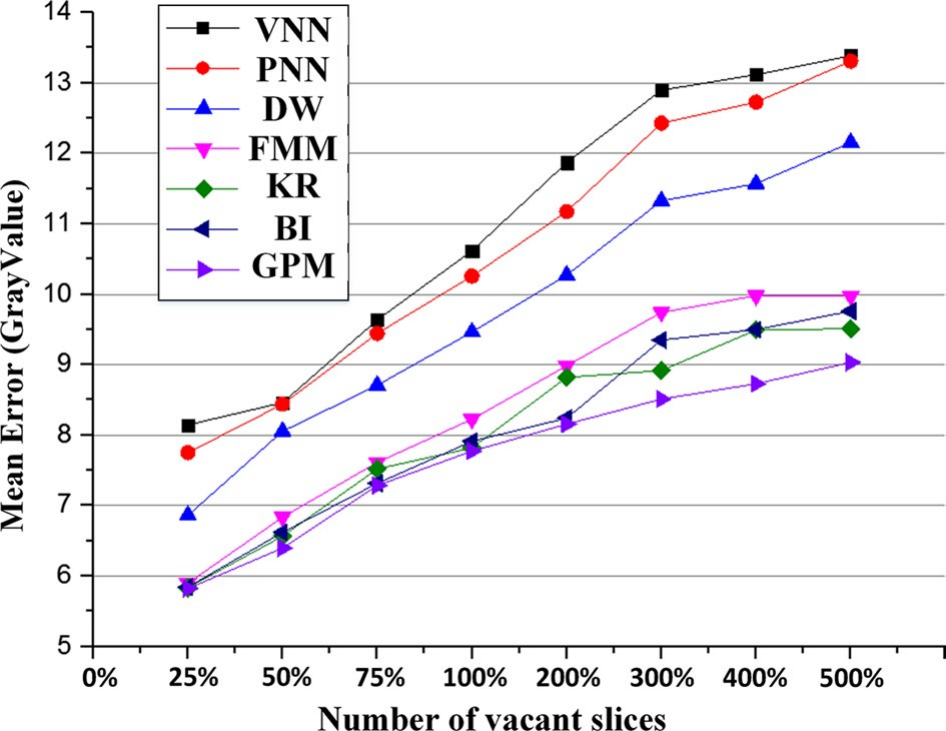
Comparing of the five reconstruction errors with respect to different size of removing patches

To further quantify the performance of the proposed method, we randomly remove patches with the size of 25, 50, 75 and 100% of the cross-section slice from the volume data. While 200 to 500% represent removing two to five cross-sections from the volume data. Figure 6 compares the mean reconstruction error of the VNN, PNN, DW, FMM, KR, BI and GPM methods. It can be seen that the reconstruction error increase with the increase of size and number of the deleting patches. For the removing patch size of 25%, the reconstruction means errors for VNN, PNN, DW, FMM, KR, BI and GPM methods are 8.14, 7.75, 6.86, 5.90, 5.84, 5.84 and 5.82, respectively. While for the removing patch size of 500%, the reconstruction means errors for VNN, PNN, DW, FMM, KR, BI and GPM methods are 13.38, 13.31, 12.15, 9.97, 9.51, 9.76 and 9.03, respectively. It can be seen that the performances of the VNN and PNN are quite closed to each other, especially for low ratios of the removing patch. Moreover, it can be seen that DW performs better than that of the VNN and PNN methods. And the removing ratio less than 50%, the performance of the FMM and GPM are very close, but GPM achieves lower reconstruction errors than that of the FMM, KR and BI method for removing ratio larger than 50%. For all the seven methods, the proposed GPM method is the best, which can obtain the minimum reconstruction error for all the mission ratios of the volume data.

**Fig. 7:**
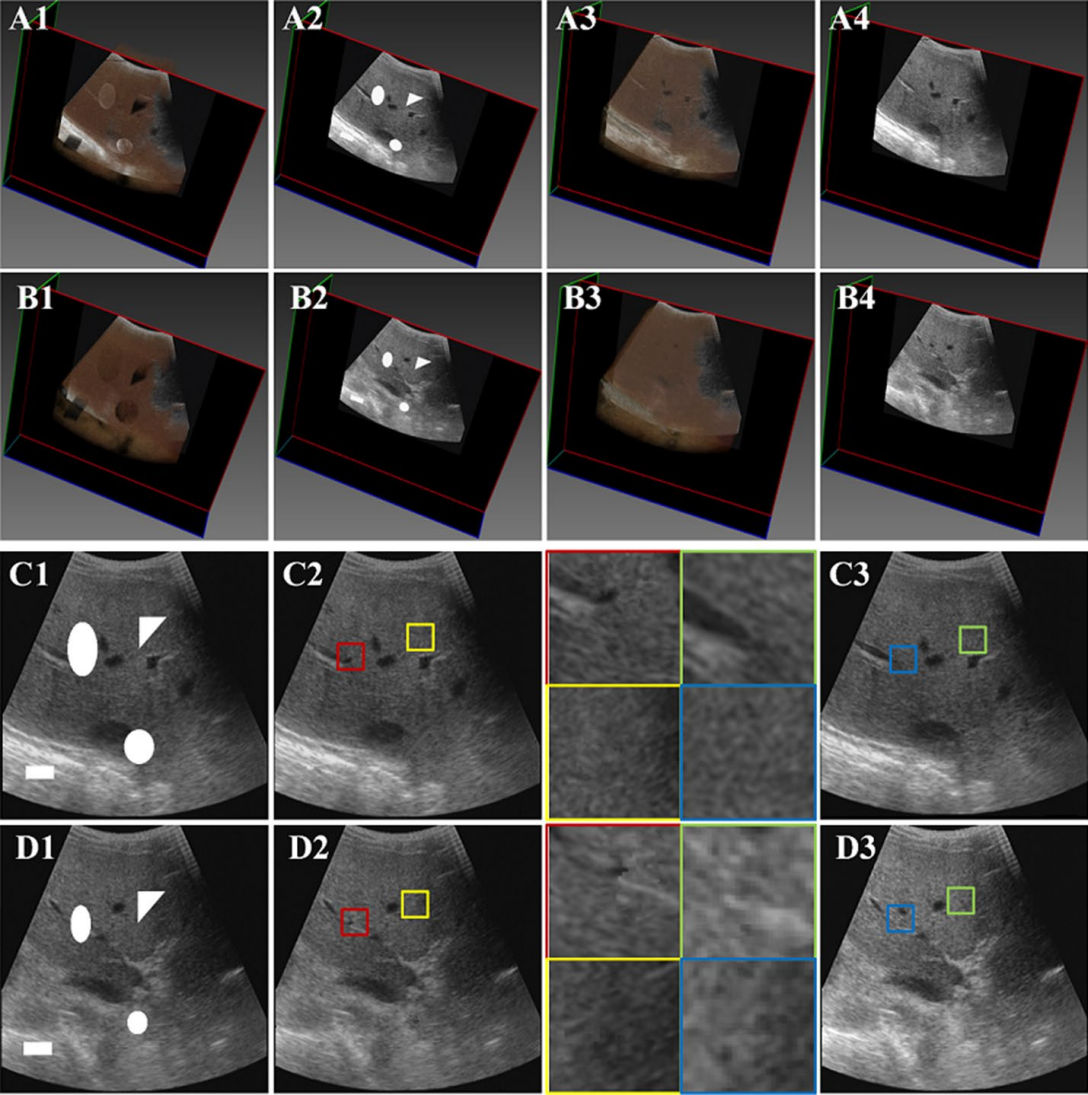
The reconstruction results with GPM method

Figure 7 shows the reconstruction results of the GPM methods. The experiments utilize data set 1 and data set 2, and three predefined sizes of volumes, including a cube, a sphere, a tetrahedron and an ellipsoid (as can be seen in Fig. 7) are removed from the original data set. Figure 7A1, B1, C1, D1 show four ultrasound slices with predesigned holes. Figure 7A2, B2, C2, D2 give the reconstruction results of the GPM methods. Figure 7A3, B3, C3, D3 give the original four ultrasound slices. The right column shows the magnified regions of interest corresponding to the second column. From the figure, it can be seen that the GPM method capable of repairing the vacant patches by similar intensities that approach to their neighboring pixels, and the reconstructed region is seamlessly merged with the neighboring pixels. Therefore, it is obvious that the proposed GPM method can produce good reconstruction results for different size or shape of vacant regions in the ultrasound volume data.

### Quantification of abnormality reconstruction results

The state and location of the lesion can be preliminarily demonstrated according to the abnormality in the ultrasonic image. Thus, the abnormalities should be reconstructed accurately to provide precise information of lesion for the clinical diagnosis. The process of vacant patch repairing is the most important step for the reconstruction. The effectiveness of the proposed GPM to reconstruct the abnormality is verified by the following experiments.

**Fig.8:**
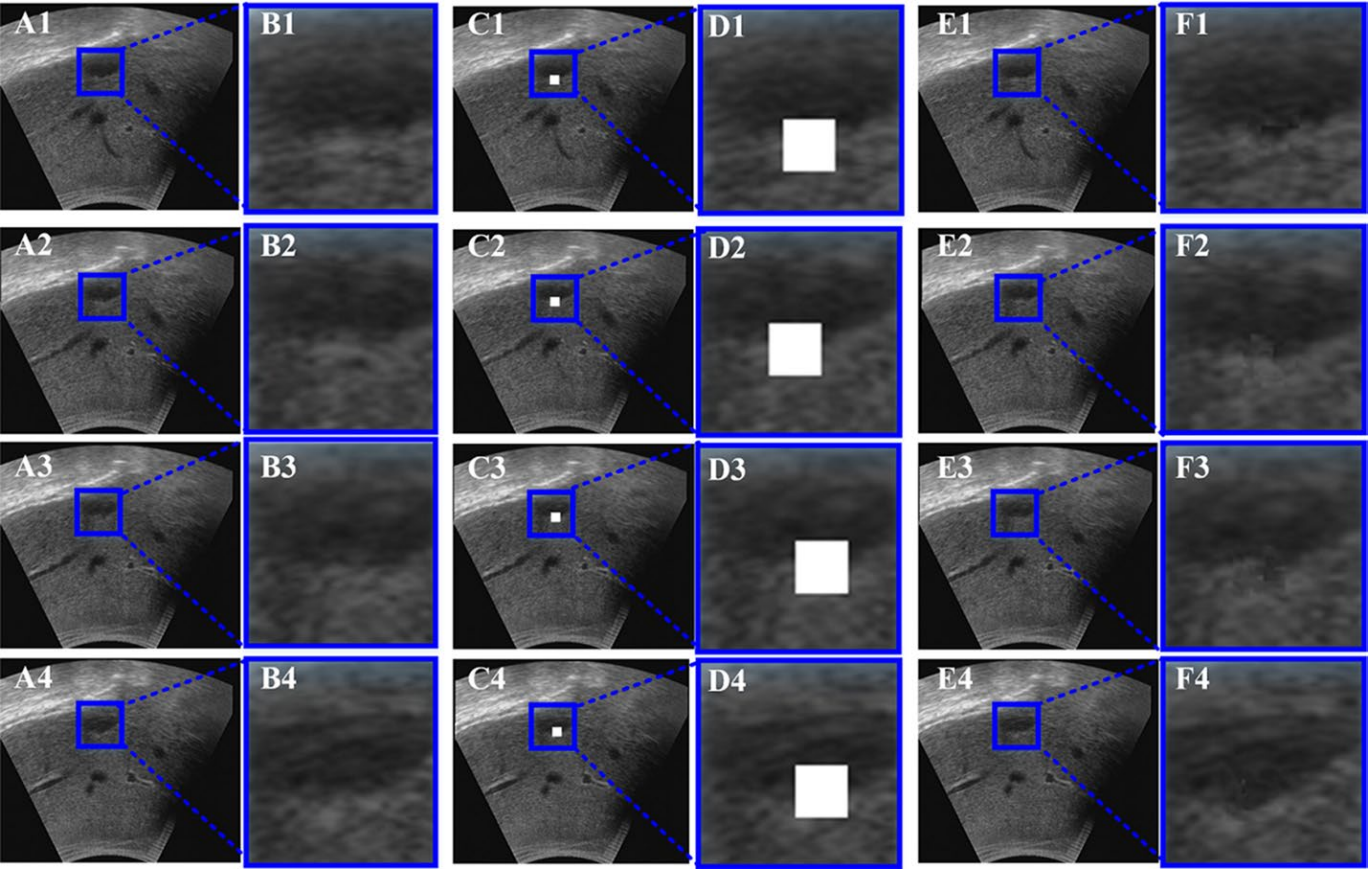
The abnormality reconstruction results with GPM method

**Fig. 9:**
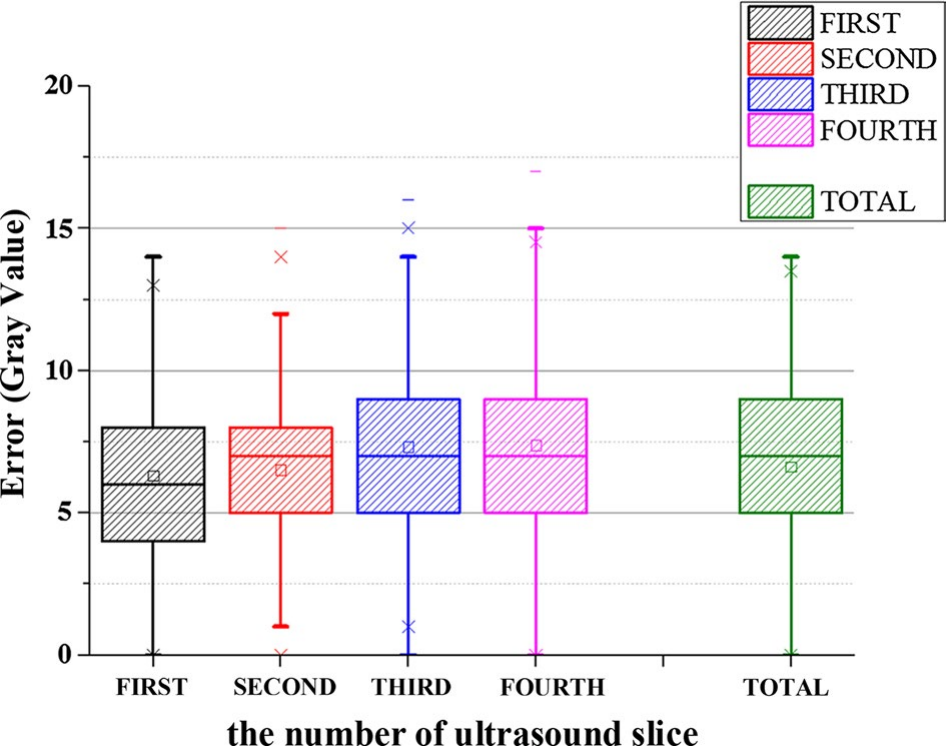
The abnormality reconstruction errors with GPM method

A predefined size of cubes (20 × 20 × 115) is removed from the edge area of the abnormality in data set 2. Figure 8A1–A4 show four slices extracted from the original ultrasound volume. And the corresponding voxel removed slices are shown in Fig. 8C1–C4. Figure 8E1–E4 show the results of the GPM methods. The corresponding details of the vacant patch area are enlarged and shown in Fig. 8B1–B4, D1–D4, F1–F4, separately. It is clear that the vacant patches can be accurately repaired and the edge of the abnormality is seamlessly merged with the neighboring pixels. No blur is caused. The reconstruction errors for each ultrasound slices and the mean reconstruction error are shown in Fig. 9. The reconstruction errors and the standard deviation are 7.154 and 7.248, respectively. The reconstruction errors of seventy-five percent are in the range of [5.028, 8.718]. The experimental results demonstrate that the abnormality can be reconstructed precisely. Furthermore, the reconstruction result is not affected by noise because the size of noises is typically one voxel but the size of a patch used to repair empty voxel is 9 × 9 × 9. The noise has no effect on the repairing calculation and the best matching patch selection. Therefore, it is obvious that the proposed GPM method can produce good reconstruction results for the abnormality regions in the ultrasound volume data.

### Computation complexity analysis

The efficiency is crucial for the 3D freehand ultrasound reconstruction in clinical usage. To perform an objective evaluation of the computation time, let *O* represents the computational complexity of the algorithm,$N_{u} ,N_{v}$ represent the dimensions of the B-scan slices in $u,v$ direction. $N_{x} ,N_{y} ,N_{z}$ represent the dimensions of the volume grid in $x,y,z$ direction. $N_{p}$ represents the number of the acquired 2D b-scan images, $R$ is the size of the spherical interpolation region. *T* is the time for optimizing the parameters of Bezier curve. Table 4 lists the computational complexity of VNN, PNN, DW, FMM, KR, BI and the proposed GPM methods. From the pseudo-code described in [15], it is noticeable that the VNN, PNN, DW, FMM and KR approaches traverse each voxel to assign voxel value. Thus, the loop number can be utilized to represent the total number of the reconstructed volume grid. Moreover, the VNN, DW and BI approaches require finding the shortest distance to each B-scan for each voxel in each loop. As the size of sampled B-scans is usually several hundreds, such shortest-distance-finding processes in the inner loop hence may dramatically increase the computation time. While the loop number of FMM interpolation algorithm is $O(M \cdot \log (M) \cdot R)$ (as can be seen in Ref. [50, 51]).

**Table 4:** Computational time complexity for VNN, PNN, DW, FMM, KR, BI and GPM algorithms

Method	Computational time complexity	
	Bin-filling scheme	Hole-filling strategy
VNN	–	$O(N \cdot N_{p} \cdot N_{u} \cdot N_{v} )$
PNN	$O(N_{p} \cdot N_{u} \cdot N_{v} )$	$O(N \cdot R)$
DW	–	$O(N \cdot N_{p} \cdot R \cdot N_{u} \cdot N_{v} )$
FMM	$O(N_{p} \cdot N_{u} \cdot N_{v} )$	$O(M \cdot \log (M) \cdot R^{3} )$
KR	$O(N_{p} \cdot N_{u} \cdot N_{v} )$	$O(M \cdot R^{6} )$
BI	–	$O(N \cdot N_{p} \cdot R \cdot N_{u} \cdot N_{v} \cdot T)$
GPM	$O(N_{p} \cdot N_{u} \cdot N_{v} )$	$O(N \cdot R \cdot N)$

$$N = N_{x} \cdot N_{y} \cdot N_{z}$$, $$M = \hbox{max} (N_{x} ,N_{y} ,N_{z} )$$

Table 5 shows the average computational times (in seconds) for VNN, PNN, DW, FMM, KR, BI and GPM algorithms in the process of 3D ultrasound volume reconstruction of data set 1 and 2. It can be seen that the computational time of the VNN algorithm is 115.0 s, which is the fastest among all the testing method. The fast computational efficiency of the VNN algorithm come from that it only needs to traverse the whole ultrasound volume once according to the space distribution of the pixels in ultrasound slices. The computational time of the DW algorithm is 263.6 s because that the construction process of DW algorithm includes the Bin-filling scheme and the Hole-filling strategy. After the bin-filling scheme, there are a lot of holes and empty voxels in the 3D volume data and the neighboring voxels are utilized to fill the empty voxels. The computational time of the KR and BI algorithm are 2937.0 and 2163.4 s, respectively. It takes a long time to optimize the parameters of kernel and Bezier curve. The computational time of the GPM algorithm is 240.95 s, which is also with large time burden. The reason is that the hole filling strategy of GPM algorithm is to fill the vacant voxels in the 3D ultrasound volume data through finding the best matching patch in the whole 3D ultrasound volume data.

**Table 5:** Computational time for VNN, PNN, DW, FMM, KR, BI and GPM algorithms

Method	Data set 1			Data set 2			Average
	Bin-filling scheme	Hole-filling strategy	Total	Bin-filling scheme	Hole-filling strategy	Total	
VNN	–	98.6	98.6	–	131.4	131.4	115.00
PNN	33.8	66.3	100.1	54.6	93.7	148.3	124.20
DW	–	203.5	203.5	–	323.7	323.7	263.60
FMM	39.1	62.4	101.5	55.7	83.8	139.5	120.50
KR	34.9	2491.5	2524.4	55.1	3294.4	3349.5	2937.0
BI	–	1715.5	1715.5	–	2611.3	2611.3	2163.4
GPM	43.5	145.6	189.1	61.1	231.7	292.8	240.95

Unit: second

## Discussion

3D ultrasound volume reconstruction from B-model ultrasound slices can provide clear and intuitive images for tissues and lesions for the clinician, which has very important value in clinical practice. This paper proposed a novel global patch matching method for the reconstructing of 3D ultrasound volume from a series of free-hand B-model ultrasound slices. The proposed reconstruction method includes two main steps: bin-filling scheme and 3D hole-filling strategy. The bin-filling scheme is aimed to fill and interpolate the 3D volume data by the obtained ultrasound slices. To fully utilized the orientation information of each ultrasound slice and suppress the interference of the noise, we introduce the median absolute deviation the inter-quartile ranges absolute deviation to calculate the invariant property of each pixel of ultrasound image. Based on invariant response of the existing ultrasound images, the optimum contribution range for the overlapping range can be obtained. Then, the intensity of overlapping region can be obtained by weighted combination of the pixels within the best contribution ranges. The hole-filling strategy is designed to fill holes and gaps in the 3D volume data according to the texture and intensity distribution of the existing ultrasound voxels. For this method, the priority of the filling order of the vacant region is calculated by optimizing the confidence term, the data term and the gradient term. Then, hole-filling process starts from the voxel with the maximum priority weights. And the filling patch is obtained by finding the patch with maximum similarity measures in the whole volume data.

The performance of the proposed algorithm is evaluated on both phantom data and in vivo ultrasound data sets. For these data sets, we randomly delete a series of predefined holes and a number of continuous ultrasound slices, as the grand truths of the ultrasound volumes are exactly known, the reconstruction accuracy of the algorithms can be effectively quantified. The proposed algorithm is compared with the other four up to date reconstruction methods, including VNN, PNN, DW, FMM, KR, BI. From the experiments of the filled hole and ultrasound reconstruction, it can be seen that there are apparent boundaries for the results of VNN and PNN methods. The results may stem from both the VNN and PNN methods that utilize nearest neighboring pixels for the interpolation of the hole region, and the interpolated region usually has the same intensity values. And the intensity distribution in the interpolated region presents homogeneous property, which hence leads to obvious boundaries between the filled hole and its neighboring region. Compared with VNN and PNN methods, there are no obvious boundaries for the filled and the neighboring regions for the DW and BI method. As the DW and BI method utilizes a considerable number of pixels for the interpolation, the filled region seems very fuzzy and the details cannot be clearly identified. The filled region of the FMM and KR method seems clearer than that of the DW method. It is because that the FMM and BI method constraint the interpolation sequence, which hence effectively improves the sharpness of the filled region. Compared with the other methods, it can be seen that the proposed GPM method not only effectively preserves the detail of the filling region, but also, the filled regions are naturally mixed with the neighboring pixels. There are no distinct boundaries for the filling regions. Experiments demonstrate that the proposed method is very effective and robust for reconstructing ultrasound images. Among all the comparing methods, the proposed GPM method can restore the 3D ultrasound volume with minimum difference.

## Conclusion

For the proposed GPM method, the overlapping pixel is determined by the neighboring pixels within the best contribution range, which hence can effectively utilize the neighboring similar pixels and maintain the smoothness of the interpolated voxels. And the vacant voxels are filled by searching the most similar patches in the whole volume data. Hence, the volume data is filled with high reconstruction accuracy and effectively preserves homogeneous distribution of the textures. For each vacant voxel, the proposed GPM method needs to search the best matching patch in the whole existing volume data, which hence is time consuming. The future study will be focused on GPU based accelerating of the reconstruction algorithm.

## Abbreviations

GPM: Global Patch Matching
PNN: pixel nearest neighbor
VNN: voxel nearest neighbor
DW: distance weighted
FMM: fast marching method
KR: kernel regression
BI: Bezier interpolation
RBF: radial basis function
MAD: median absolute deviation
IQRAD: inter-quartile range absolute deviation
PVA-C: polyvinyl alcohol cryogel
